# Sex-specific progression of Parkinson's disease: A longitudinal mixed-models analysis

**DOI:** 10.1177/1877718X251339201

**Published:** 2025-05-19

**Authors:** Anne-Marie Hanff, Christopher McCrum, Armin Rauschenberger, Gloria A Aguayo, Claire Pauly, Sonja R Jónsdóttir, Olena Tsurkalenko, Maurice P Zeegers, Anja K Leist, Rejko Krüger, on behalf of the NCER-PD consortium

**Affiliations:** 1 Transversal Translational Medicine, Luxembourg Institute of Health, Strassen, Luxembourg; 2 Translational Neurosciences, Luxembourg Centre for Systems Biomedicine, University of Luxembourg, Esch-sur-Alzette, Luxembourg; 3 Department of Epidemiology, CAPHRI Care and Public Health Research Institute, Maastricht University Medical Centre+, Maastricht, The Netherlands; 4 Department of Nutrition and Movement Sciences, NUTRIM School of Nutrition and Translational Research in Metabolism, Maastricht University Medical Centre+, Maastricht, The Netherlands; 5 Biomedical Data Science, Luxembourg Centre for Systems Biomedicine, University of Luxembourg, Esch-sur-Alzette, Luxembourg; 6 Bioinformatics and Artificial Intelligence, Department of Medical Informatics, Luxembourg Institute of Health, Strassen, Luxembourg; 7 Department of Precision Health, Luxembourg Institute of Health, Strassen, Luxembourg; 8 Parkinson Research Clinic, Centre Hospitalier de Luxembourg, Luxembourg, Luxembourg; 9 Digital Medicine group, Luxembourg Institute of Health, Strassen, Luxembourg; 10 Department of Epidemiology, Care and Public Health Research Institute, School of Nutrition and Translational Research in Metabolism, Maastricht University, Maastricht, the Netherlands; 11 Department of Social Sciences, University of Luxembourg, Esch-sur-Alzette, Luxembourg

**Keywords:** neurodegenerative disease, patient reported outcome measures, epidemiology, cohort studies, women, sex differences

## Abstract

**Background:**

Despite its relevance, the clinical progression of motor- and non-motor symptoms associated with Parkinson's disease (PD) is poorly described and understood, particularly in relation to sex-specific differences in clinical progression.

**Objective:**

Identification of differential aspects in disease progression in men and women with PD.

**Methods:**

Linear mixed-model analyses of 802 people with typical PD from the Luxembourg Parkinson's study's prospective cohort (median time of follow-up = three years). We estimated the effect of time and its moderation by sex (alpha ≤ 0.05), including confidence intervals, for the following outcomes: MDS-UPDRS I-IV, Starkstein Apathy Scale, Beck Depression Inventory, Montreal Cognitive Assessment (MoCA), Sniffin’ sticks, bodily discomfort, rapid eye movement sleep behavior disorder questionnaire, PD Sleep Scale (PDSS), Munich Dysphagia Test-PD, Functional Mobility Composite Score, and the MDS-based tremor and postural instability and gait disturbances scale. In addition, the marginal means illustrated the symptoms’ trajectories in men and women. Men and women had similar age.

**Results:**

Overall, we observed a slower progression (interaction effect) in women compared to men, especially for MoCA (−0.159, 95%CI [−0.272, −0.046], p = 0.006), PDSS (−0.716, 95%CI [−1.229, −0.203], p = 0.006), PIGD (0.133, 95%CI [0.025 0.241], p = 0.016), and MDS-UPDRS II (0.346, 95%CI [0.120, 0.572], p = 0.003). The finding for MDS-UPDRS II was significant (FWER of 5%) after adjustment for multiple comparisons (Bonferroni-Holm).

**Conclusions:**

Next to the further exploration of sex-specific progression, interventions, proactive monitoring and communication strategies tailored to the symptoms progression and needs of men and women need to be developed.

## Plain language summary

Although Parkinson's disease is 1.4 times more frequent in men compared to women, it is not clear how the symptoms develop over time in men and women. Consequently, we aimed to describe the changes of different symptoms over time in both sexes and to find out if women with Parkinson's disease showed a different progression than men. By analyzing repeated measurements of 802 individuals, we found an overall slower worsening of the symptoms in women compared to men, particularly for memory, sleep, walking and balance as well as motor symptoms in general. Consequently, compared to women, men not only experience Parkinson's disease more frequently but also experience a faster worsening of symptoms. More research is needed to fully understand why. To improve the well-being of men and women with Parkinson's disease, we suggest taking into account the important differences between them when doing research and providing health care.

## Introduction

In the 2016 Global Burden of Disease Study, the age-standardized prevalence of Parkinson's disease (PD) was 1.4 times higher in men than in women.^1^ This male preponderance might be explained by a protective effect of female sex hormones, a different genetic mechanism or different exposures to environmental risk factors in men and women.^2^ Consequently, sex-specific factors in PD merit further study. However, most research has focused on biological differences between men and women, neglecting to place these in the psychosocial context that impacts clinical care and quality of life of men and women with PD.^3–5^ Therefore, the effect of sex and/or gender should be considered in designing future studies in PD.^6^

Moreover, previous longitudinal studies addressed the sex-specific progression of some symptoms. Thus, the association of sex with patient-reported and clinician-assessed motor symptoms, the phenotype, activities of daily living and medication with progression was investigated.^7^ Another study^8^ explored the role of sex in the progression of patient-reported motor symptoms, cognition, dyskinesia, wearing off, depression, rapid eye movement (REM) sleep behavior disorder and some non-motor symptoms. However, most often single studies^3–5^ have mainly reported cross-sectional sex differences of selected symptoms in men and women with PD while a comprehensive empirical description and illustration of the motor- and non-motor symptoms associated with PD progression has not been reported in the literature. Aiming to provide an overview of symptom and general disease progression of PD in men and women that can be easily interpreted by health professionals, we describe the progression of motor- and non-motor symptoms in men and women and quantify the effect moderation by sex in people with typical PD participating in a large monocentric longitudinal cohort.

## Methods

### Study design, setting, participants, and study size

This retrospective analysis is part of the Luxembourg Parkinson's study, a nationwide, monocentric, observational, longitudinal-prospective and dynamic cohort.^9,10^ The completed STROBE reporting guideline checklist^11^ is included in Supplemental Table 3.

All participants underwent diagnostic evaluation and were assigned a clinical diagnosis of typical PD or Parkinson's disease dementia (PDD) by a neurologist based on established United Kingdom Parkinson's Disease Society Brain Bank Clinical Diagnostic Criteria.^12^ The diagnosis was not required before participation as the Luxembourg Parkinson's study also included converters. The participants were recruited from Luxembourg and the Greater Region (geographically close areas of the surrounding countries, namely Belgium, France, and Germany). In addition to the referral by medical doctors, a communication campaign (advertisement on radio and television, dedicated webpage, social media campaign, multilingual flyers and posters, fact sheets and bi-annual print newsletter, collaboration with patient associations) informed the population about the option to enroll themselves. Recruitment started in 2015 with annual follow-ups. The Luxembourg Parkinson's Study aims at stratification and differential diagnosis of PD.^9,10^

### Variables, data sources, and measurement

The outcomes of interest were progression (i.e., change per additional year since diagnosis) of motor and non-motor symptoms. Patient-reported outcomes included: apathy, measured by the Starkstein Apathy Scale (SAS); depression, assessed using the Beck Depression Inventory (BDI-I); dysphagia, assessed by the Munich Dysphagia Test for PD (MDT-PD); functional mobility, evaluated by the PDQ-39 based functional mobility composite score (FMCS); non-motor symptoms, captured by the Movement Disorders Society-Unified Parkinson's Disease Rating Scale Part I (MDS-UPDRS I); motor symptoms, assessed by MDS-UPDRS II; bodily discomfort, measured using the respective subscale of the Parkinson's Disease Questionnaire-39 (PDQ-39); quality of sleep, evaluated with the Parkinson's Disease Sleep Scale (PDSS); rapid eye movement (REM) sleep behavior disorder, screened using the REM sleep Behavior Disorder (RBD) Screening Questionnaire (RBDSQ). Clinician-assessed outcomes and performance tests included global cognition, assessed using the Montreal Cognitive Assessment (MoCA); motor symptoms evaluated by the MDS-UPDRS III; motor complications, measured by MDS-UPDRS IV; olfaction, tested using the Sniffin’ Sticks Identification Test; Postural Instability and Gait Disturbances (PIGD), assessed using the MDS-UPDRS-based PIGD score; tremor, evaluated using the MDS-UPDRS-based tremor scale. Table 1 describes the characteristics of the outcomes and provides sources of data and details of the assessment methods. All outcomes were numerical and assessed during annual follow-ups varying by a maximum of three months to minimize seasonal influences. The progression could be distinguished from cohort or period effects as people with PD were included at different time points^13^ due to the dynamic cohort study design. People with PD with complete data for time since diagnosis were included in the longitudinal analysis.

**Table 1.** Instrument, assessment types and variable name of the included constructs.

Construct intended to measure	Instrument	Assessment type	Variable name
*Patient-reported outcomes*			
Apathy	SAS ^34^	Patient-Reported Outcome Measure	spark_score
Depression	BDI-I ^35^	Patient-Reported Outcome Measure	bdi_score
Dysphagia	MDT-PD ^36,37^	Clinician- Assessed Outcome Measure	mdt_score
Functional mobility	FMCS ^38^	Patient-Reported Outcome Measure	FMCS_PDQ39
Non-motor symptoms	MDS-UPDRS I ^39^	Patient-Reported and Clinician Assessed Outcome Measure	UPDRS_1
Motor symptoms	MDS-UPDRS II ^39^	Patient-Reported Outcome Measure	UPDRS_2
Bodily discomfort	PDQ-39 subscale bodily discomfort ^40^	Patient-Reported Outcome Measure	pdq39_q36_q39_score
Quality of sleep	PDSS ^41^	Patient-Reported Outcome Measure	pdss_score
Rem-sleep behavior disorder	RBDSQ ^42^	Patient-Reported Outcome Measure	rem_score
*Clinician assessed outcomes or performance tests*			
Cognition	MoCA Total Score ^43^	Performance test	MoCA_score
Motor symptoms	MDS-UPDRS III ^39^	Clinician-Assessed Outcome Measure	UPDRS_3
Motor fluctuations	MDS-UPDRS IV ^39^	Clinician-Assessed Outcome Measure	UPDRS_4
Olfaction	ODOFIN Sniffin’ Sticks Identification Test 16	Performance test	sniff_score
Postural instability and gait disturbance	MDS-UPDRS based PIGD score ^44,45^,	Patient-Reported and Clinician Assessed Outcome Measure	PIGD_score
Tremor	MDS-UPDRS based tremor scale ^45,46^	Patient-Reported and Clinician Assessed Outcome Measure	trem_trem_score
*Exposure*			
Time variant with baseline assessment and yearly follow-up	Disease duration (y.): Date of assessment – Date of diagnosis	Interview	disease_duration
*Confounder*			
Time variant with baseline assessment and yearly follow-up	Time to diagnosis (y.): Date of diagnosis – Date of first motor symptoms	Interview	diagnosis_duration

BDI-I: Beck Depression Inventory I; FMCS: Functional Mobility Composite Score; MDS: Movement Disorders Society; MDT: Munich Dysphagia Test; MoCA: Montreal Cognitive Assessment; PDQ39: Parkinson's Disease Questionnaire; PDSS: Parkinson's Disease Sleep Scale; PIGD: Postural Instabilities and Gait Disturbances; RDBSQ: RBD Screening Questionnaire; SAS: Starkstein Apathy Scale; UPDRS: Unified Parkinson's Disease Rating Scale.

### Statistical methods

Data analysis was carried out in R, version 4.3.1.^14^ We used the two-sided Wilcoxon rank-sum test for discrete variables and the chi-squared (χ^2^) test for categorical variables compared baseline characteristics between men and women (using the “stats” package^14^). In addition to the Bonferroni-adjusted p-values (p-value * 30 variables ≤ 0.05) we provided the unadjusted p-values (p-value ≤ 0.05).

**Figure 1.**
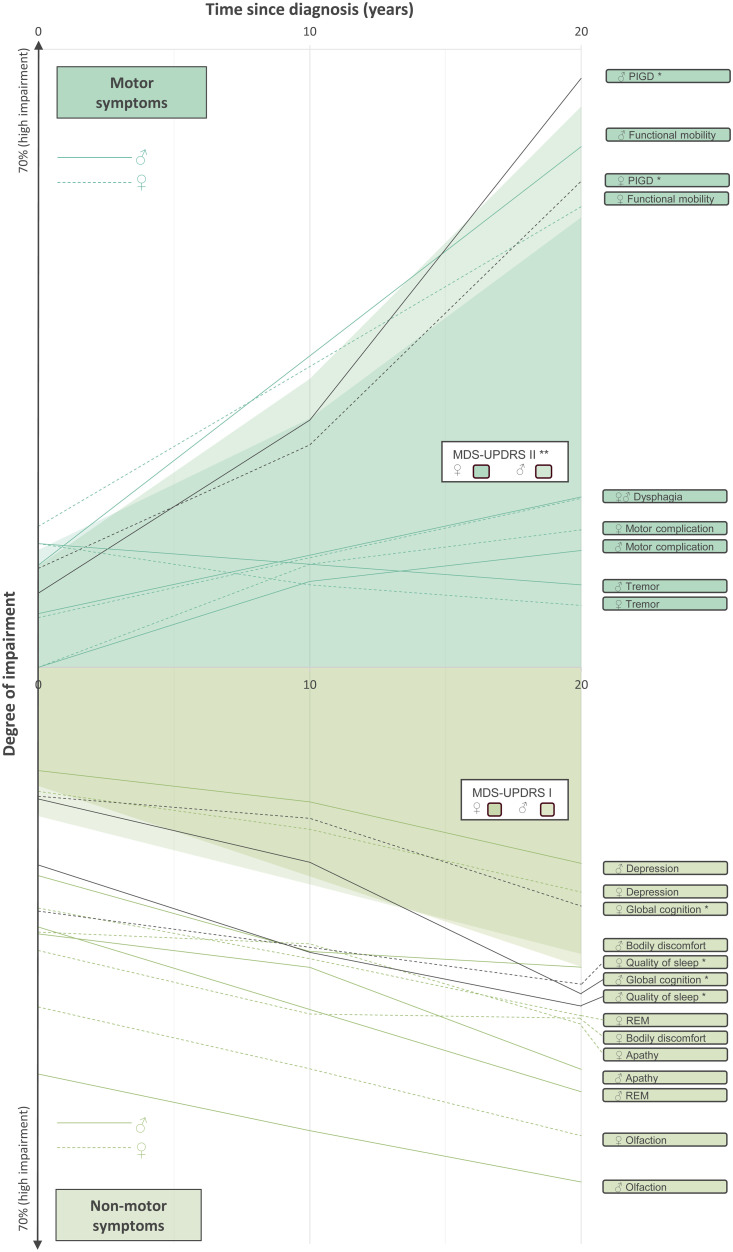
Progression of motor- and non-motor symptoms in men and women with typical PD 0-20 years after diagnosis. Degree of impairment = 0–100% (greater = worse). * = nominally significant, ** significant after adjustment for FWER 5% (Bonferroni-Holm), lines of significant results are highlighted in black. PD: Parkinson's disease; PIGD: postural instabilities and gait disturbances; RBD: rapid eye movement (REM) behavior disorder.

To describe the progression of different motor- and non-motor symptoms and the effect moderation by sex, we created one model per outcome (using “lmer”-function of the “lme4”-package^15^). Consequently, we performed longitudinal two-level mixed models analyses with fixed effects for years since diagnosis and sex, a random intercept on participant level and a random slope for years since diagnosis. In addition to the linear effect we tested whether adding a quadratic effect of time significantly improved the fit of the model. If this was the case, we additionally tested the cubic effect of time. Difference in progression between men and women was described by a significant interaction effect for sex and time on the symptoms. We estimated the linear mixed models using the maximum likelihood method while statistical significance and confidence intervals for the mixed models were obtained with the Kenward-Roger approximation for degrees of freedom. We adjusted the p-values using the Bonferroni-Holm procedure to maintain the Family-Wise Error Rate (FWER) at 5%. To enhance clinical interpretation, we provided estimated marginal means, i.e., estimated means of motor- and non-motor symptoms given 0, 10, and 20 years since diagnosis. Thus, we examined the range of the years since diagnosis from its minimal observed value to its maximal observed value, then fixed the covariates (diagnosis duration) at their mean to finally look at the estimated values for the different symptoms for the whole range of values of years since diagnosis (using “ggpredict”-function of the “ggeffects”-package^16^). Those estimated means for the different symptoms (y-axis) given years since diagnosis (x-axis) and the mean value for the covariates were illustrated as an interaction plot (using the “plot_model”- function of the “sjPlot”-package^17^). As women's ratings of disability differed between self-reported and physician-reported,^18^ we categorized the results in patient-reported or clinician-assessed outcomes / performance tests. Time, in this case modelled as years since diagnosis, was included in the mixed models to describe progression of the different outcomes (significance tested via t-test). Degree of disability as illustrated in Figure 1 was calculated by the following formula:

$$Degree\;of\;disability=\left(\frac{Estimated\;marginal\;means}{Maximum\;score}\times 100\right).$$

For illustrative purposes in Figure 1, the following scores were inverted to the higher, the worse: functional mobility (FMCS), quality of sleep (PDSS), global cognition (MoCA), and olfaction (Sniffin’ Sticks). Also, we focused in the figure from data the first twenty years after diagnosis as most observations were collected in that period.

## Results

**Figure 2.**
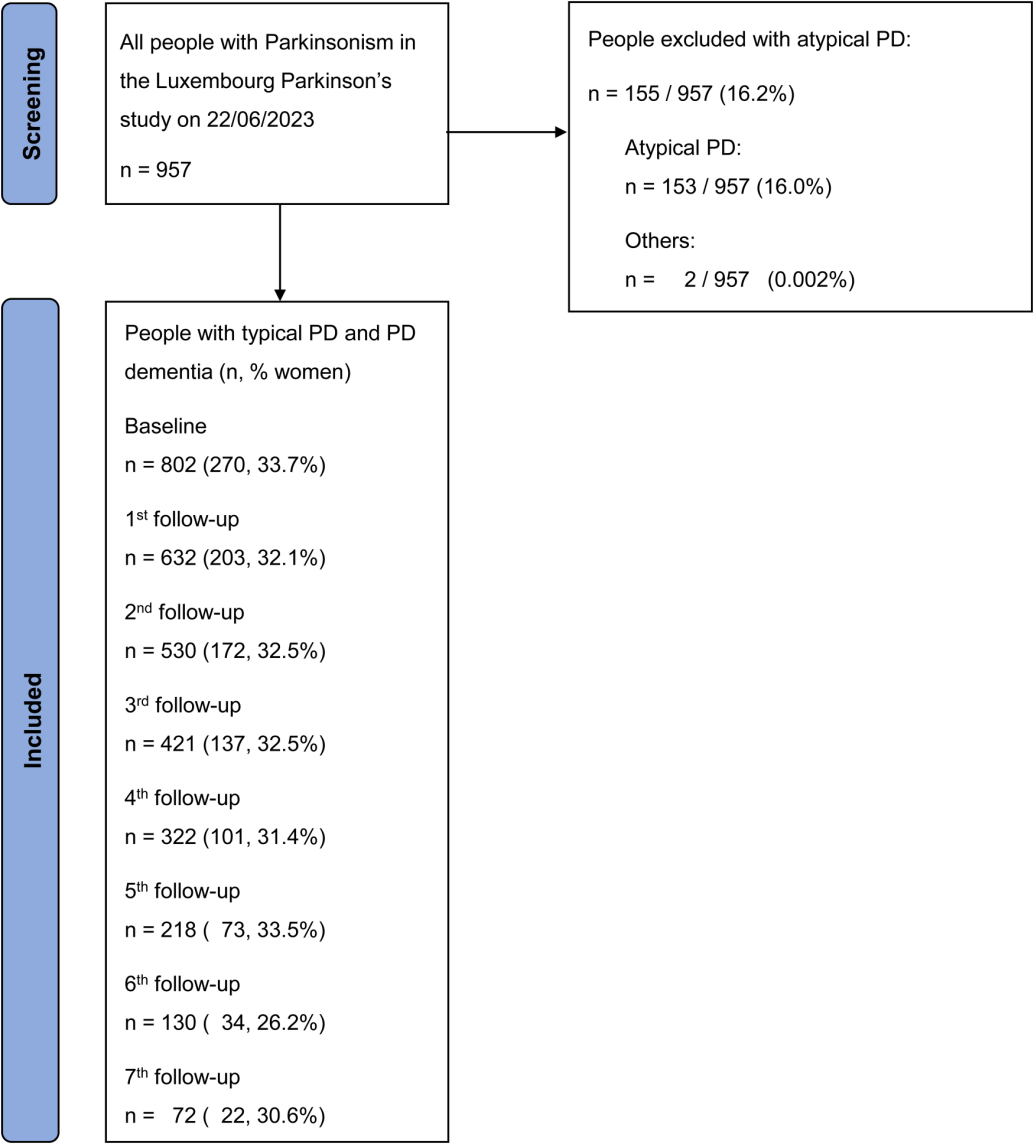
Flow diagram of recruitment.

As illustrated in Figure 2, 957 persons participated in the Luxembourg Parkinson's Study up to the date of data export (2023-06-22). After the exclusion of people with atypical PD, we included 802 people with typical PD with a baseline assessment between 04.03.2015 and 22.06.2023.

Table 2 summarizes key study characteristics to understand the potential applicability, and thus generalizability of the findings, while Supplemental Table 1 provides a detailed description of the study participants and missing data. The clinical and demographic characteristics of study participants at baseline by sex are presented in Table 3. Testing for differences at baseline in 30 characteristics at a Bonferroni-adjusted 5% significance level, women had worse scores for depression (Beck Depression Inventory I - BDI-I) than men, with a median difference of 1.99 (95% CI [0.99, 2.99]), W = 74343, p = 0.006. Also, women reported worse scores for bodily discomfort (PDQ-39 subscale bodily discomfort) than men, with a median difference of 8.3 (95% CI [0.000006, 8.33]), W = 74468, p = 0.004. However, women had better olfaction scores (Sniffin’ sticks) than men with a median difference of 1.0 (95% CI [0.99, 1.99]), W = 74969, p < 0.001. Finally, women had a higher Levodopa Equivalent Daily Dose (LEDD) per kg of body weight (mg/ kg) than men with a median difference of 1.2 mg/kg (95% CI [0.5, 1.9]), W = 57869, p = 0.02 and a lower body mass index (BMI) than men with a median difference of 2.0 kg/m^2^ (95% CI [−2.7, −1.3]), W = 50270, p < 0.001. Also, women had less years of education (median difference = 1 (95% CI [−1.9, −0.9]), W = 58475, p = 0.004) and experienced a bereavement more often than men, χ^2^ (df = 2, N = 797) = 48.81, p < 0.001. We did not identify any statistically significant differences for age, years since diagnosis or time to diagnosis at baseline between men and women with typical PD. Missing data patterns were visually inspected for sociodemographic characteristics and the different outcomes; most variables had missing data for less than 5% of the male and female samples. Rates for missing data were higher for Munich Dysphagia Test-assessed dysphagia (51% and 55% for men and women, respectively).

**Table 2.** Key characteristics.

*Sample size*	802
*Data collection period*	04.03.2015–22.06.2023
*Study design*	Cohort
*Average number of observations*	3.0 (3.0)
*Setting*	People with typical PD living at home or in a nursing home in Luxembourg and the greater region
*Inclusion criteria*	People with typical PD
*Sex*	269 (33.6%) women
*Age*	68.2 (14.3)
*Disease stage*	2.0 (0.5)
*Outcomes* Concept (Measure)	Apathy (SAS), depression (BDI-I), functional mobility (FMCS), height (cm), LEDD (mg/kg), non-motor symptoms (MDS-UPDRS I), patient-reported motor symptoms (MDS-UPDRS II), clinician-assessed motor symptoms (MDS-UPDRS III), motor complications (MDS-UPDRS IV), dysphagia (MDT-PD), global cognition (MoCA), olfaction (Sniffin’ Sticks), bodily discomfort (PDQ-39 subscale bodily discomfort), health-related quality of life (PDQ-39), quality of sleep (PDSS), postural instabilities and gait disturbances (MDS-based PIGD), REM sleep behavior disorder (RBDSQ), tremor (MDS-based tremor scale), weight (kg)
*Determinants*	Disease duration, time to diagnosis

Categorical variables: counts (%), numerical variables: Median (IQR). PD: Parkinson's disease; BDI-I: Beck Depression Inventory; FMCS: Functional Mobility Composite Score; LEDD: Levodopa Equivalent Daily Dose; MDS: Movement Disorders Society; MDT: Munich Dysphagia Test; MoCA: Montreal Cognitive Assessment; PDQ39: Parkinson's Disease Questionnaire; PDSS: Parkinson's Disease Sleep Scale; PIGD: Postural Instabilities and Gait Disturbances; RBDSQ: RBD Screening Questionnaire; SAS: Starkstein Apathy Scale; UPDRS: Unified Parkinson's Disease Rating Scale.

**Table 3.** Characteristics of men and women.

Variables	Men (N = 532)	Women (N = 270)	Unadjusted p	Adjusted p
*S* *ociodemographic characteristics*				
Age (y)	68.2 (14.5)	68.1 (14.3)	p = 0.393	p = 1
Years of education	13.0 (4.0)	12.0 (4.8)	p < 0.001	p = 0.004
Most fluently spoken language			p = 0.715	p = 1
Luxembourgish	234 (44.0%)	111 (41.1%)		
French	145 (27.3%)	82 (30.4%)		
German	84 (15.8%)	45 (16.7%)		
Other	69 (13.0%)	31 (11.5%)		
Children (n)	2.0 (2.0)	2.0 (1.0)	p = 0.009	p = 0.256
Marital status			p < 0.001	p < 0.001
Single	20 (3.8%)	24 (8.9%)		
Married / Partnered	442 (83.1%)	164 (60.7%)		
Divorced / Bereaved	67 (12.6%)	80 (29.6%)		
*Health-rel* *ated characteristics*				
Diagnosis			p = 0.167	p = 1
PD	463 (87.0%)	244 (90.4%)		
PDD	69 (13.0%)	26 (9.6%)		
Hoehn and Yahr (H&Y) Disease Stages			p = 0.356	p = 1
H&Y 1	58 (10.9%)	30 (11.1%)		
H&Y 1.5	43 (8.1%)	26 (9.6%)		
H&Y 2	275 (51.7%)	119 (44.1%)		
H&Y 2.5	64 (12.0%)	41 (15.2%)		
H&Y 3	45 (8.5%)	31 (11.5%)		
H&Y 4	27 (5.1%)	13 (4.8%)		
H&Y 5	11 (2.1%)	5 (1.9%)		
Phenotype			p = 0.004	p = 0.120
Tremor dominant	223 (41.2%)	84 (31.1%)		
Intermediate	58 (10.9%)	24 (8.9%)		
PIGD dominant	198 (37.2%)	129 (47.8%)		
Disease duration (y.)	3.1 (5.9)	3.5 (6.6)	p = 0.108	p = 1
Age at diagnosis (y.)	63.0 (16.5)	63.0 (17.0)	p = 0.297	p = 1
Age at onset of motor-symptoms (y.)	61.0 (18.0)	60.0 (17.2)	p = 0.212	p = 1
Time to diagnosis (y.)	1.0 (3.0)	1.0 (3.0)	p = 0.549	p = 1
LEDD (mg/kg)	5.5 (6.1)	6.6 (7.7)	p = 0.001	p = 0.024
PDQ-39 (0–100)a	19.9 (22.4)	25.0 (21.6)	p = 0.004	p = 0.127
Body Mass Index (BMI)	27.7 (5.2)	25.9 (6.4)	p < 0.001	p < 0.001
Weight (kg)	83.7 (18.7)	65.5 (17.4)	not tested	not tested
Height (cm)	173.7 (11.0)	161.0 (9.6)	not tested	not tested
*Non-mot* *or symptoms*				
MoCA (0–30)^b^	25.0 (5.0)	26.0 (5.0)	p = 0.012	p = 0.370
BDI-I (0–63)^a^	8.0 (9.0)	10.0 (9.0)	p < 0.001	p = 0.007
SAS (0–42)^a^	13.0 (7.0)	13.0 (7.0)	p = 0.335	p = 1
Sniffin’ Sticks (0–16)^b^	8.0 (5.0)	9.0 (4.0)	p < 0.001	p < 0.001
PDQ-39 subscale bodily discomfort (0–100)^a^	25.0 (33.3)	41.7 (41.7)	p < 0.001	p = 0.004
PDSS (0–150)^b^	110.0 (34.0)	106.5 (36.1)	p = 0.020	p = 0.592
RBDSQ (0–13)^a^	4.0 (5.0)	4.0 (4.0)	p = 0.178	p = 1
MDS-UPDRS I (0–52)^a^	9.0 (8.0)	10.0 (9.0)	p = 0.012	p = 0.354
*M* *otor symptoms*				
MDS-UPDRS II (0–52)^a^	10.0 (10.0)	9.0 (10.0)	p = 0.744	p = 1
MDS-UPDRS III (0–132)^a^	33.0 (21.0)	31.0 (23.8)	p = 0.194	p = 1
MDS-UPDRS IV (0–24)^a^	0.0 (0.0)	0.0 (2.5)	p = 0.172	p = 1
FMCS (0–100)^b^	81.2 (31.2)	76.6 (34.4)	p = 0.038	p = 1
MDS-UPDRS based PIGD Score (0–20)^a^	2.0 (4.0)	3.0 (4.0)	p = 0.062	p = 1
MDS-UPDRS based tremor Scale (0–4)^a^	0.5 (0.6)	0.5 (0.6)	p = 0.029	p = 0.882
MDT Score (3–103)^a^	6.0 (9.0)	6.0 (7.8)	p = 0.467	p = 1

Categorical variables: counts (%), numerical variables: median (IQR), a : Greater = worse, b : Greater = better, numerical variables : two-sided Wilcoxon rank-sum test, categorical variables : chi-squared test. PD: Parkinson's disease; PDD: PD dementia; BDI-I: Beck Depression Inventory I; FMCS: Functional Mobility Composite Score; LEDD: Levodopa Equivalent Daily Dose; MDS: Movement Disorders Society; MDT: Munich Dysphagia Test; MoCA: Montreal Cognitive Assessment; PDQ39: Parkinson's Disease Questionnaire; PDSS: Parkinson's Disease Sleep Scale; PIGD: Postural Instabilities and Gait Disturbances; RBDSQ: RBD Screening Questionnaire; SAS: Starkstein Apathy Scale; UPDRS: Unified Parkinson's Disease Rating Scale.

While many outcomes showed a linear trajectory, this was not the case for apathy (SAS), global cognition (MoCA), depression (BDI-I), bodily discomfort (PDQ-39 subscale bodily discomfort), patient-reported motors symptoms (MDS-UPDRS II), motor complications (MDS-UPDRS IV), postural instability and gait disturbances (MDS-UPDRS based PIGD score) where adding the quadratic effect significantly improved the fit of the model. We described the model statistics and the detailed fixed and random effects in Tables 4 and 5 and illustrated the progression (estimated marginal means converted to % impairment) of men and women in Figure 1. Supplemental Figures 1–3 detail the interaction plots for clinical interpretation.

**Table 4.** Fixed effects of non-motor symptoms in men and women and the interaction of sex with progression.

	SAS^a^		MoCA^b^		BDI-I^a^		MDS-UPDRS I^a^		Sniffin’ sticks^b^		PDQ-39 subscale bodily discomfort^a^		RBDSQ^a^		PDSS^b^	
Independent variables	B (CI 95%)	p	B (CI 95%)	p	B (CI 95%)	p	B (CI 95%)	p	B (CI 95%)	p	B (CI 95%)	p	B (CI 95%)	p	B (CI 95%)	p
Intercept	13.501 (12.499 – 14.502)	<0.001	25.314 (24.661 – 25.967)	<0.001	9.401 (8.148 – 10.654)	<0.001	9.269 (8.198 – 10.340)	<0.001	9.353 (8.831 – 9.875)	<0.001	34.586 (30.675 – 38.497)	<0.001	3.629 (3.104 – 4.154)	<0.001	105.934 (102.021 – 109.846)	<0.001
Years since diagnosis	−0.105 (−0.275 – 0.065)	0.225	0.051 (-0.076 – 0.178)	0.435	0.164 (-0.048 – 0.377)	0.129	0.437 (0.300 – 0.573)	<0.001	-0.119 (−0.171 – −0.066)	<0.001	1.141 (0.521 – 1.761)	<0.001	0.085 (0.023 – 0.147)	0.007	−0.668 (−1.086 – −0.251)	0.002
Years since diagnosis^2	0.017 (0.009 – 0.025)	<0.001	−0.013 (−0.018 – −0.007)	<0.001	0.011 (0.001 – 0.021)	0.026	-	-	-	-	−0.037 (−0.063 – −0.010)	0.007	-	-	-	-
Time to diagnosis	−0.012 (−0.088 – 0.064)	0.748	−0.008 (−0.062 – 0.047)	0.787	0.025 (−0.062 – 0.113)	0.574	0.034 (−0.049 – 0.116)	0.420	0.008 (−0.032 – 0.047)	0.708	−0.075 (−0.357 – 0.208)	0.605	0.046 (0.005 – 0.088)	0.029	−0.103 (−0.397 – 0.191)	0.492
Male sex	0.108 (−1.015 – 1.232)	0.850	−0.061 (−0.792 – 0.669)	0.869	−1.561 (-2.968 – −0.153)	0.030	−1.823 (-3.093 – −0.552)	0.005	−1.276 (−1.898 – −0.655)	<0.001	-9.125 (−13.518 – −4.732)	<0.001	0.303 (−0.320 – 0.925)	0.340	7.039 (2.391 – 11.687)	0.003
Years since diagnosis: Male sex	0.110 (−0.037 – 0.257)	0.143	−0.159 (−0.272 – −0.046)	0.006*	−0.031 (−0.211 – 0.149)	0.733	0.130 (−0.038 – 0.297)	0.129	0.013 (−0.053 – 0.078)	0.706	0.148 (−0.354 – 0.650)	0.562	0.045 (−0.031 – 0.121)	0.245	−0.716 (−1.229 – −0.203)	0.006*
*Random effects*																
σ^2^	9.39		1006.93		14.58		13.71		3.23		182.41		2.52		203.06	
τ_00_	24.83 _ND_		1711.10 _ND_		40.99 _ND_		31.43 ND		7.62 _ND_		366.48 _ND_		8.33 _ND_		417.36 _ND_	
τ_11_	0.19 _ND.disease_duration_		2.40 _ND.disease_duration_		0.33 _ND.disease_duration_		0.29 _ND.disease_duration_		0.01 _ND.disease_duration_		0.90 _ND.disease_duration_		0.05 _ND.disease_duration_		0.91 _ND.disease_duration_	
ρ_01_	−0.34 _ND_		1.00 _ND_		−0.54 _ND_		−0.45 ND		−0.57 _ND_		−0.35 _ND_		−0.43 _ND_		−0.45 _ND_	
ICC	0.75		0.72		0.72		0.72		0.66		0.66		0.77		0.64	
N	739 _ND_		633 _ND_		738 _ND_		751 ND		718 _ND_		746 _ND_		736 _ND_		741 _ND_	
Observations	2623		1664		2519		2832		2319		2669		2590		2617	
Marginal R^2^ / Conditional R^2^	0.091 / 0.777		0.029 / 0.727		0.084 / 0.748		0.146 / 0.761		0.059 / 0.680		0.045 / 0.670		0.045 / 0.777		0.068 / 0.667	

*nominally significant, **significant after adjustment for FWER 5% (Bonferroni-Holm), ^a^Greater: Worse; ^b^ Greater: Better; Β: fixed effect (95% CI); MoCA: Montreal Cognitive Assessment; SAS: Starkstein Apathy Scale; BDI-I: Beck Depression Inventory; MDS: Movement Disorders Society; UPDRS: Unified Parkinson's Disease Rating Scale; PDQ-39: Parkinson's Disease Questionnaire-39; PDSS: Parkinson's Disease Sleep Scale; RBDSQ: RBD Screening Questionnaire.

**Table 5.** Fixed effects of motor symptoms in men and women and the interaction of sex with progression.

	FMCS^b^		MDS-UPDRS II^a^		MDS-UPDRS III^a^		MDS-UPDRS IV^a^		MDS-UPDRS PIGD score^a^		MDS-UPDRS based tremor scale^a^		MDT-PD^a^	
Independent variables	B (CI 95%)	p	B (CI 95%)	p	B (CI 95%)	p	B (CI 95%)	p	B (CI 95%)	p	B (CI 95%)	p	B (CI 95%)	p
Intercept	83.082 (79.747 – 86.417)	<0.001	7.269 (6.090 – 8.448)	<0.001	26.671 (24.355 – 28.987)	<0.001	0.054 (−0.321 – 0.429)	0.776	2.400 (1.888 – 2.911)	<0.001	0.537 (0.464 – 0.609)	<0.001	6.379 (4.551 – 8.206)	<0.001
Years since diagnosis	−1.943 (−2.450 – −1.435)	<0.001	0.607 (0.370 – 0.844)	<0.001	1.072 (0.743 – 1.401)	<0.001	0.383 (0.287 – 0.479)	<0.001	0.121 (0.006 – 0.236)	0.039	−0.014 (−0.023 – −0.005)	0.002	0.730 (0.481 – 0.978)	<0.001
Years since diagnosis^2			0.022 (0.012 – 0.032)	<0.001			−0.009 (−0.014 – −0.004)	<0.001	0.017 (0.012 – 0.022)	<0.001				
Time to diagnosis	−0.060 (−0.345 – 0.224)	0.676	0.043 (−0.056 – 0.143)	0.394	0.147 (−0.032 – 0.327)	0.107	−0.000 (−0.025 – 0.025)	0.998	0.008 (−0.034 – 0.051)	0.695	0.007 (0.001 – 0.012)	0.019	0.110 (−0.030 – 0.249)	0.124
Male sex	4.720 (0.777 – 8.663)	0.019	−0.957 (−2.277 – 0.362)	0.155	2.910 (0.174 – 5.645)	0.037	−0.260 (−0.665 – 0.146)	0.209	−0.718 (−1.287 – −0.149)	0.013	0.056 (−0.029 – 0.142)	0.196	−0.472 (−2.625 – 1.681)	0.667
Years since diagnosis:Male sex	−0.597 (−1.217 – 0.023)	0.059	0.346 (0.120 – 0.572)	0.003**	0.197 (−0.206 – 0.600)	0.338	−0.018 (−0.101 – 0.065)	0.668	0.133 (0.025 – 0.241)	0.016*	0.004 (−0.007 – 0.015)	0.467	0.014 (−0.289 – 0.318)	0.927
*Random effects*														
σ^2^	81.89		14.35		84.54		4.93		3.23		0.07		28.92	
τ_00_	341.79 _ND_		30.49 _ND_		111.92 _ND_		0.00 _ND_		4.25 _ND_		0.14 _ND_		66.95 _ND_	
τ_11_	6.42 _ND.disease_duration_		0.78 _ND.disease_duration_		1.36 _ND.disease_duration_		0.08 _ND.disease_duration_		0.16 _ND.disease_duration_		0.00 _ND.disease_duration_		0.74 _ND.disease_duration_	
ρ_01_	−0.38 _ND_		−0.10 _ND_		−0.24 _ND_				0.05 _ND_		−0.38 _ND_		−0.23 _ND_	
ICC	0.88		0.85		0.67				0.83		0.68		0.78	
N	742 _ND_		755 _ND_		753 _ND_		753 _ND_		751 _ND_		753 _ND_		675 _ND_	
Observations	2645		2877		2536		2904		2500		2503		2077	
Marginal R^2^ / Conditional R^2^	0.190 / 0.900		0.346 / 0.904		0.145 / 0.717		0.182 / NA		0.335 / 0.889		0.034 / 0.691		0.107 / 0.800	

*nominally significant; **significant after adjustment for FWER 5% (Bonferroni-Holm). ^a^Greater: Worse; ^b^ Greater: Better; Β: fixed effect (95% CI); FMCS: Functional Mobility Composite Score; MDS: Movement Disorders Society; UPDRS: Unified Parkinson's Disease Rating Scale; PIGD: Postural Instabilities and Gait Disturbances; MDT: Munich Dysphagia Test.

Women overall demonstrated a slower progression than men. More specifically, men had a significantly faster progression in global cognition (MoCA) (r = −0.159, 95% CI [−0.272, −0.046], p = 0.006, Table 4), quality of sleep (PDSS) (r = −0.716, 95% CI [−1.229, −0.203], p = 0.006, Table 4) and postural instabilities and gait disturbance (MDS-based PIGD score) (r = 0.133, 95% CI [0.025, 0.241], p = 0.016, Table 5) on an unadjusted significance level and in patient-reported motor symptoms (MDS-UPDRS II) (r = 0.346, 95% CI [0.120, 0.572], p = 0.003, Table 5). The findings for patient-reported motor symptoms were significant after adjustment for FWER 5%. After controlling for age in the model analyzing the progression in global cognition, the p-value for the interaction effect between sex and time on global cognition decreased from 0.006 to 0.004 while we did not identify any statistically significant differences for age, years since diagnosis or time to diagnosis at baseline between men and women with typical PD. Finally, the frequency of missing data at follow-up in women was not significantly higher than in men.

## Discussion

The present study described and illustrated the progression of motor- and non-motor symptoms in men and women with typical PD. Both men and women showed a progression (i.e., deterioration) in all symptoms. Comparing symptoms progression between men and women, women experienced a slower progression in global cognition (MoCA), quality of sleep (PDSS), postural instabilities and gait disturbances (MDS-UPDRS based PIGD score) and patient-reported motor symptoms (MDS-UPDRS II). Finally, we observed similar trajectories for patient-reported outcomes compared to clinician-assessed outcomes in both men and women.

### Non-motor symptoms

Previous reviews^3–5^ discussed the heterogeneous findings of sex-specific progression of PD. According to our findings, women tended to have a generally slower disease progression than men. As women had a slower progression of PIGD and patient-reported motor symptoms, the worse bodily discomfort at baseline in women compared to men (similar to previous findings^18–21^) might be experienced in relation to something else, e.g., different symptom expressions, such as the restless legs syndrome being more common and severe in women,^21^ while this sex-specific effect moderation was not identified in the longitudinal data. Similarly, in women, depression (BDI-I) was worse at baseline while no sex-specific effect moderation was identified in the longitudinal data, similar to previous research.^7^

Our study confirmed that after similar scores at baseline, women's cognitive performance (MoCA) declined slower over time.^8^ Moreover, we observed a similar progression of apathy (SAS), a feature of PD dementia.^22^ Interestingly, women had a similar age and frequency of PD dementia and had even less years of education than men. Consequently, future research should consider examining reserves (e.g., hormones, different genetic mechanism or different exposures to environmental risk factors) protecting women from a decline. Finally, while women had a worse quality of sleep (PDSS) at baseline, we detected a faster progression in men than compared to women.

### Motor symptoms

Our results support previous longitudinal findings^7,8^ of women having higher disability scores at baseline, but men progressing faster. We did not detect any significant differences in the motor complications (MDS-UPDRS IV) while in women the mg/kg LEDD dose was significantly higher compared to men at baseline. As we included the LEDD (mg per kg body weight), i.e., taking into account the systematic differences in weight and height between men and women, our findings might not be comparable to previous findings.^7^ Our results also confirm previous findings^6^ that the PIGD dominant phenotype is more frequent in women. However, we cannot confirm previous findings^23^ describing impairments in global cognition were associated with more severe PIGD symptoms, as despite the more frequent PIGD dominant phenotype at baseline, women had slower cognitive decline (MoCA). As only half of the phenotypes remain stable over three years^24^ and postural instabilities and gait disturbances (PIGD) progressed slower in women, this finding needs to be further explored. We used assessment instruments as recommended by the Movement Disorders Society.^25–27^ While the sex-specific effect moderation was not significant for the clinician-assessed motor symptoms (MDS-UPDRS III), we found a significant sex-specific effect moderation for patient-reported motor symptoms (MDS-UPDRS II), warranting further investigation into the underlying reasons for this divergence (e.g., sex-specific validity, differences in how women and men experience or report motor symptoms). In addition to patient-reported motor symptoms (MDS-UPDRS II), PIGD and quality of sleep (PDSS), female sex was also associated with a slower progression of global cognition (MoCA). As global cognition could be associated with the experience of patient-reported outcomes, the role of global cognition as a covariate needs to be taken into account by future studies interested in the direct effect of sex on disease progression. Finally, our study confirms recent findings^28^ of tremor severity decreasing over time. Further analysis about the difference between resting, postural and kinetic tremor are required.

### Strengths and limitations

This study has some strengths and limitations. For instance, we enhanced the generalizability of our findings by analyzing data of all participants of the Luxembourg Parkinson's study including people with PD or PDD from Luxembourg and the Greater Region, who were treated and lived in varying settings and environments. More specifically, the range of people with PD was broad, including men and women from 22 to 92 years with 1 to 30 years of education, living from 0 to 32 years with the disease and speaking different languages. 68.7% of the people with PD were in disease stages H&Y 1–2, the disease stages ranged from H&Y 1 to H&Y 5. Recruitment started in 2015 when the estimated prevalence of PD in Luxembourg was 565–1356.^9,10^ As 486 of the participants lived in Luxembourg, this corresponds to an estimated coverage of 35.8 to 86.0% of the people with PD living in Luxembourg.

Moreover, we used advanced statistical methodology to estimate changes over time in our longitudinal dataset with mixed models taking into account the dependence between repeated measures. Although our analysis is observational, our longitudinal study provided a comprehensive description of the individual progression of symptoms in PD while previous studies were mainly cross-sectional analyses with some exceptions.^7,29–31^ Most observations refer to the first twenty years since diagnosis. Consequently, our results should not be interpreted beyond this period. Future research interested in the detailed symptoms trajectories could explore alternative non-linear models, such as asymptotic functions, which might better detail the long-term trajectories of motor complications. Also, the COVID-19 pandemic and deaths since baseline assessment (101, 12.6%) led to missing data. For the Munich Dysphagia Test (MDT) score, we noted higher rates of missing values, as it was added later during the study explaining the nature of the missing values. Consequently, the analyses on this outcome should be considered exploratory. Despite the potential sampling bias for the analyses involving the onsite test MDS-UPDRS III, the frequency of missing data at follow-up was similar in both groups. Data collection standards have been developed to minimize missing data and information bias.

Our research described the progression since the diagnosis and is applicable to the progression in the first twenty years. Future research should use data of risk and prodromal cohorts to describe the biological progression before the diagnosis of PD.^32^ Our study focused on describing sex-specific progression. In the current study, women were characterized by fewer years of education, a higher likelihood of being divorced or widowed, a lower BMI, a higher LEDD (mg/kg), more symptoms of depression (BDI-I) and bodily discomfort (PDQ-39 subscale) but better olfaction (Sniffin’ sticks). Consequently, future research investigating the direct effects of sex could consider these variables along with relevant comorbidities or other factors (e.g., hearing loss, hormones, different genetic mechanisms or exposures to environmental risk factors) as covariates. Moreover, the biological plausibility for the sex-specific progression in PD and the sociocultural and behavioral protective factors in women need to be further investigated. In addition, health professionals should adapt their communication and non-pharmacological disease management strategies to the different needs of men and women (e.g., sex-specific communication strategies, flyers, and exercises). Specifically, in men during the first ten years after diagnosis, communication and interventions should focus on the promotion of global cognition, patient-reported motor symptoms (MDS-UPDRS II), quality of sleep (PDSS) and postural instabilities and gait disturbances (PIGD)^33^ while information and interventions targeting women could focus on promoting bodily comfort (e.g., prevent muscle cramps, joint pain, unpleasant hot/cold sensation) and mental health.

In conclusion, our study provided a comprehensive data-based description and illustration of the clinical progression of motor- and non-motor symptoms associated with PD for men and women. Moreover, the detailed interaction plots should aid interpretation by health professionals. Factors explaining the resilience in women with PD especially in global cognition, quality of sleep, patient-reported motor symptoms and postural instability and gait disturbances need to be explored further. To enhance well-being and personalized treatment in PD, interventions, proactive monitoring and communication strategies tailored to the symptoms progression in men and women need to be developed.

## Supplemental Material

sj-docx-1-pkn-10.1177_1877718X251339201 - Supplemental material for Sex-specific progression of Parkinson's disease: A longitudinal mixed-models analysis Supplemental material, sj-docx-1-pkn-10.1177_1877718X251339201 for Sex-specific progression of Parkinson's disease: A longitudinal mixed-models analysis by Anne-Marie Hanff, Christopher McCrum, Armin Rauschenberger, Gloria A Aguayo, Claire Pauly, Sonja R Jónsdóttir, Olena Tsurkalenko, Maurice P Zeegers, Anja K Leist, Rejko Krüger and in Journal of Parkinson's Disease
