# Supplementary material for: Sex-specific progression of Parkinson's disease: A longitudinal mixed-models analysis
Source: J Parkinsons Dis. 2025 May 19:1877718X251339201. Online ahead of print. doi: 10.1177/1877718X251339201 (PMC13347478; doi:10.1177/1877718X251339201)
Supplement: Supplementary file 1 [file sj-docx-1-pkn-10.1177_1877718X251339201.docx]

# Supplemental Material

Sex-specific progression of Parkinson’s disease: A longitudinal mixed-models analysis

**Supplemental Figure 1.** Progression of functional mobility (A), patient-reported motor symptoms (B), dysphagia (C), and pain (D), estimated marginal means, (95% CI) 0 – 20 years after the diagnosis, a Greater = Worse, b Greater = Better, red = women, blue = men

**Supplemental Figure 2.** Progression of quality of sleep (E), depression (F), apathy (G), non-motor symptoms (H) and REM sleep behavior disorder (I), estimated marginal means (95% CI) 0 – 20 years after the diagnosis, a Greater = Worse, b Greater = Better, Lines in panel F = Cutoff for mild (10 - 18), moderate (19 - 29) or severe (> 29) depression, red = women, blue = men

**Supplemental Figure 3.** Progression of cognition (J), olfaction (K), postural instabilities and gait disturbances (L), tremor (M), clinician assessed motor symptoms (N) and motor complications (O), estimated marginal means (95% CI) 0 – 20 years after the diagnosis, a Greater = Worse, b Greater = Better, Lines in panel J = Cutoff for mild (21 - 25) and severe (< 21) cognitive impairment, red = women, blue = men

**Supplemental Table 1.** Characteristics of the study participants at baseline (N = 802) including numbers of missing data for each variable of interest

| **Characteristics** | **Mean (SD)/ n (%)** | **Min. - Max.** | **Median (Pct25-75)** | **Missing N (%)** |
| --- | --- | --- | --- | --- |
| **Sociodemographic characteristics** | |  |  |  |
| Age (y) | 67.1 (10.9) | 22.0 – 92.9 | 68.2 (60.2 – 74.5) | 1 (0.1%) |
| Female Sex | 270 (33.7%) |  |  | 0 (0.0%) |
| Years of Education | 13.0 (4.1) | 1.0 – 30.0 | 13.0 (10.0 - 16.0) | 9 (1.1%) |
| Language most fluent |  |  |  | 1 (0.1%) |
| French | 227 (28.3%) |  |  |  |
| German | 129 (16.1%) |  |  |  |
| Luxembourgish | 345 (43.0%) |  |  |  |
| Other | 100 (12.5%) |  |  |  |
| Marital status |  |  |  | 5 (0.6%) |
| Single | 44 (5.5%) |  |  |  |
| Married / Partnered | 606 (75.6%) |  |  |  |
| Divorced / Bereaved | 147 (18.3%) |  |  |  |
| **Health-related characteristics** | |  |  |  |
| PD Diagnosis | 707 (88.2%) |  |  | 0 (0%) |
| Hoehn & Yahr (H&Y) Disease Stages |  |  |  | 14 (1.7%) |
| H&Y 1 | 88 (11.0%) |  |  |  |
| H&Y 1.5 | 69 (8.6%) |  |  |  |
| H&Y 2 | 394 (49.1%) |  |  |  |
| H&Y 2.5 | 105 (13.1%) |  |  |  |
| H&Y 3 | 76 (9.5%) |  |  |  |
| H&Y 4 | 40 (5.0%) |  |  |  |
| H&Y 5 | 16 (2.0%) |  |  |  |
| Disease Duration (y) | 5.0 (5.1) | 0.0 – 32.3 | 3.2 (1.1 - 7.4) | 54 (6.7%) |
| LEDD (mg.) | 493.4 (400.4) | 0.0 – 2062.0 | 400.0 (200.0 – 712.8) | 24 (3.0%) |
| LEDD (mg./kg.) | 7.3 (5.4) | 0.0 – 36.9 | 5.8 (3.6 – 10.0) | 34 (4.2%) |
| Time to Diagnosis (y.) | 2.7 (5.1) | -1.0 – 46.0 | 1.0 (0.0 – 3.0) | 30 (3.7%) |
| Weight (kg) | 79.2 (16.4) | 40.1 – 153.0 | 78.5 (67.7 – 89.4) | 21 (2.6%) |
| Height (cm) | 169.3 (9.7) | 137.0 – 205.0 | 169.1 (162.2 – 176.2) | 25 (3.1%) |
| **Non-motor symptoms** |  |  |  |  |
| MoCA (0 – 30)^b^ | 24.6 (4.2) | 5.0 – 30.0 | 25.0 (23.0 - 28.0) | 22 (2.7%) |
| BDI-I (0 – 63)^a^ | 9.8 (7.3) | 0.0 – 51.0 | 8.0 (5.0 - 14.0) | 46 (5.7%) |
| SAS (0 - 42)^a^ | 14.0 (5.9) | 1.0 – 36.0 | 13.0 (10.0 – 17.0) | 54 (6.7%) |
| PDQ-39 (0 – 100)^a^ | 24.6 (17.3) | 0.0 – 82.1 | 21.8 (10.9 – 34.6) | 69 (8.6%) |
| MDT Score (3 - 103)^a^ | 8.7 (9.2) | 0.0 – 56.0 | 6.0 (3.0 – 11.0) | 375 (46.8%) |
| Sniffin’ Sticks (0 - 16)^b^ | 8.1 (3.2) | 1.0 – 16.0 | 8.0 (6.0 – 10.0) | 60 (7.5%) |
| PDQ-39 Subscale Bodily Discomfort (0 – 100)^a^ | 33.2 (23.9) | 0.0 – 100 | 33.3 (16.7 – 50.0) | 44 (5.5%) |
| PDSS (0 - 150)^b^ | 105.4 (24.9) | 17.0 – 150.0 | 108.4 (90.3 – 125.0) | 59 (7.4%) |
| RBDSQ (0 - 13)^a^ | 4.5 (3.2) | 0.0 – 13.0 | 4.0 (2.0 – 7.0) | 64 (8.0%) |
| MDS-UPDRS I (0 – 52)^a^ | 10.4 (6.9) | 0.0 – 39.0 | 9.0 (5.0 - 14.0) | 33 (4.1%) |
| **Motor symptoms** |  |  |  |  |
| MDS-UPDRS II (0 – 52)^a^ | 11.0 (8.4) | 0.0 – 48.0 | 9.0 (5.0 - 15.0) | 24 (3.0%) |
| MDS-UPDRS III (0 – 132)^a^ | 34.1 (16.7) | 0.0 – 100.0 | 32.0 (22.0 - 44.0) | 21 (2.6%) |
| MDS-UPDRS IV (0 – 24)^a^ | 1.6 (3.2) | 0.0 – 16.0 | 0.0 (0.0 - 1.0) | 17 (2.2%) |
| FMCS (0 – 100)^b^ | 74.6 (23.0) | 0.0 – 100.0 | 81.2 (60.9 - 93.8) | 46 (5.7%) |
| PIGD Score (0 – 20)^a^ | 3.5 (3.8) | 0.0 – 20.0 | 2.0 (1.0 – 5.0) | 25 (3.1%) |
| Tremor Scale (0 - 4)^a^ | 0.6 (0.4) | 0.0 – 2.4 | 0.5 (0.3 – 0.8) | 21 (2.6%) |

^a^ Greater = Worse, ^b^ Greater = Better

**Supplemental Table 2.** Estimated marginal means (95%CI) 0, 10, and 20 years after diagnosis for patient-reported and clinician-assessed outcomes and performance tests

| **Patient-reported outcomes** | | | | | | |  |
| --- | --- | --- | --- | --- | --- | --- | --- |
| **Y.** | **Apathy** SAS (0 - 42)^a^ | | **Depression** BDI-I (0 – 63)^a^ | | **Dysphagia** MDT-PD (3 – 103)^a^ | | |
|  | m | w | m | w | m | w | |
| 0 | 13.6 (12.9, 14.3) | 13.5 (12.5, 14.4) | 7.9 (7.0, 8.8) | 9.5 (8.3, 10.7) | 6.2 (5.0, 7.4) | 6.7 (4.9, 8.5) | |
| 10 | 15.3 (14.7, 16.0) | 14.1 (13.2, 15.0) | 10.3 (9.6, 11.1) | 12.4 (11.2, 13.2) | 13.7 (12.4, 14.9) | 14.0 (12.2, 15.8) | |
| 20 | 20.5 (18.8, 22.2) | 18.2 (16.1, 20.3) | 15.0 (12.9, 17.1) | 17.2 (14.7, 19.7) | 21.1 (18.3, 23.9) | 21.3 (17.4, 25.2) | |
| **Y.** | **Functional mobility** FMCS (0 – 100)^b^ | | **Non-motor symptoms** MDS-UPDRS I (0 – 52)^a^ | | **Patient-reported motor symptoms** MDS-UPDRS II (0 – 52)^a^ | | |
|  | m | w | m | w | m | w | |
| 0 | 87.6 (85.4, 89.9) | 82.9 (79.7, 86.1) | 7.5 (6.8, 8.3) | 9.4 (8.3, 10.4) | 6.4 (5.6, 7.2) | 7.4 (6.3, 8.5) | |
| 10 | 62.2 (59.5, 65.0) | 63.5 (59.6, 67.4) | 13.2 (12.5, 13.9) | 13.7 (12.8, 14.7) | 18.2 (17.1, 19.2) | 15.7 (14.2, 17.2) | |
| 20 | 36.8 (30.9, 42.8) | 44.1 (35.6, 52.5) | 18.9 (17.4, 20.4) | 18.1 (16.0, 20.2) | 35.4 (31.6, 37.1) | 28.4 (24.9, 31.9) | |
| **Y.** | **Bodily discomfort** PDQ-39 subscale bodily discomfort (0 - 100)^a^ | | **RBD** RBDSQ (0 – 13)^a^ | | **Quality of sleep** PDSS (0 – 150)^b^ | | |
|  | m | w | m | w | m | w | |
| 0 | 25.3 (22.5, 28.0) | 34.4 (30.6, 38.2) | 4.1 (3.7, 4.4) | 3.8 (3.3, 4.3) | 114.0 (111.2, 116.9) | 105.6 (101.8, 109.4) | |
| 10 | 34.5 (32.3, 36.7) | 42.1 (39.1, 45.1) | 5.4 (5.0, 5.7) | 4.6 (4.2, 5.1) | 98.1 (95.8, 100.3) | 99.0 (96.0, 101.9) | |
| 20 | 36.4 (31.2, 41.6) | 42.6 (36.1, 49.1) | 6.7 (6.0, 7.4) | 5.5 (4.5, 6.4) | 88.3 (83.6, 93.1) | 92.3 (86.2, 98.4) | |

**Supplemental Table 2 (Continued)**

| **Clinician-assessed outcomes and performance tests** | | | | | | |
| --- | --- | --- | --- | --- | --- | --- |
| **Y.** | **Cognition**  MoCA Score (0 – 30)^b^ | | **Clinician-Assessed motor symptoms**  MDS-UPDRS III (0 – 132)^a^ | | **Motor complications**  MDS-UPDRS IV (0 – 24)^a^ | |
|  | m | w | m | w | m | w |
| 0 | 25.2 (24.8, 25.7) | 25.3 (24.7, 25.9) | 30.0 (28.5, 31.5) | 27.1 (25.0, 28.9) | -0.2 (-0.5, 0.1) | 0.1 (-0.3, 0.4) |
| 10 | 22.9 (22.4, 23.4) | 24.5 (23.8, 25.3) | 42.7 (41.0, 44.4) | 37.8 (35.5, 39.8) | 2.5 (2.2, 2.9) | 3.0 (2.5, 3.5) |
| 20 | 18.1 (16.6, 19.5) | 21.3 (19.6, 23.1) | 55.4 (51.6, 59.1) | 48.5 (43.7, 52.9) | 3.4 (2.3, 4.5) | 4.0 (2.7, 5.4) |
| **Y.** | **Olfaction**  Sniffin’ Sticks (0 – 16)^b^ | | **Postural Instabilities and Gait Disturbances**  MDS-UPDRS based PIGD score (0 - 20)^a^ | | **Tremor**  MDS-UPDRS based tremor scale (0 – 4)^a^ | |
|  | m | w | m | w | m | w |
| 0 | 8.1 (7.7, 8.5) | 9.4 (8.9, 9.9) | 1.8 (1.4, 2.1) | 2.4 (1.9, 2.9) | 0.6 (0.6, 0.7) | 0.6 (0.5, 0.6) |
| 10 | 7.0 (6.8, 7.3) | 8.2 (7.8, 8.6) | 6.0 (5.5, 6.5) | 5.4 (4.6, 6.1) | 0.5 (0.5, 0.6) | 0.4 (0.4, 0.5) |
| 20 | 6.0 (5.4, 6.5) | 6.9 (6.3, 7.7) | 14.3 (12.3, 15.1) | 11.8 (10.0, 13.5) | 0.4 (0.3, 0.5) | 0.3 (0.1, 0.4) |

^a^Greater = Worse, ^b^Greater = Better. Y: years since diagnosis, Estimated marginal effects per outcome (95% CI); BDI-I: Beck Depression Inventory; FMCS: Functional Mobility Composite Score; MDS: Movement Disorders Society; MDT: Munich Dysphagia Test; MoCA: Montreal Cognitive Assessment; PDQ39: Parkinson's Disease Questionnaire; PDSS: Parkinson's Disease Sleep Scale; PIGD: Postural Instabilities and Gait Disturbances; RBDSQ: RBD Screening Questionnaire; SAS: Starkstein Apathy Scale; UPDRS: Unified Parkinson's Disease Rating Scale

**Supplemental Table 3.** STROBE Reporting guideline

|  | | Item No | | Recommendation | Page No |
| --- | --- | --- | --- | --- | --- |
| **Title and abstract** | | 1 | | (*a*) Indicate the study’s design with a commonly used term in the title or the abstract | 1 |
|  |  |  |  | (*b*) Provide in the abstract an informative and balanced summary of what was done and what was found | 3-5 |
| **Introduction** | | | | | |
| Background/rationale | | 2 | | Explain the scientific background and rationale for the investigation being reported | 6 |
| Objectives | | 3 | | State specific objectives, including any prespecified hypotheses | 6 |
| **Methods** | | | | | |
| Study design | | 4 | | Present key elements of study design early in the paper | 7 |
| Setting | | 5 | | Describe the setting, locations, and relevant dates, including periods of recruitment, exposure, follow-up, and data collection | 7 |
| Participants | | 6 | | (*a*) Give the eligibility criteria, and the sources and methods of selection of participants. Describe methods of follow-up | 7 |
|  |  |  |  | (*b*) For matched studies, give matching criteria and number of exposed and unexposed | NA |
| Variables | | 7 | | Clearly define all outcomes, exposures, predictors, potential confounders, and effect modifiers. Give diagnostic criteria, if applicable | 7-8, 29 |
| Data sources/ measurement | | 8* | | For each variable of interest, give sources of data and details of methods of assessment (measurement). Describe comparability of assessment methods if there is more than one group | 7-8 |
| Bias | | 9 | | Describe any efforts to address potential sources of bias | 14-16 |
| Study size | | 10 | | Explain how the study size was arrived at | 10 |
| Quantitative variables | | 11 | | Explain how quantitative variables were handled in the analyses. If applicable, describe which groupings were chosen and why | 7-8 |
| Statistical methods | | 12 | | (*a*) Describe all statistical methods, including those used to control for confounding | 8-10 |
|  |  |  |  | (*b*) Describe any methods used to examine subgroups and interactions | 9 |
|  |  |  |  | (*c*) Explain how missing data were addressed | 11 |
|  |  |  |  | (*d*) If applicable, explain how loss to follow-up was addressed |  |
|  |  |  |  | (*e*) Describe any sensitivity analyses | NA |
| **Results** | | | | |  |
| Participants | | 13* | | (a) Report numbers of individuals at each stage of study—eg numbers potentially eligible, examined for eligibility, confirmed eligible, included in the study, completing follow-up, and analysed | 10-11 |
|  |  |  |  | (b) Give reasons for non-participation at each stage | 10-11 |
|  |  |  |  | (c) Consider use of a flow diagram | 26 |
| Descriptive data | | 14* | | (a) Give characteristics of study participants (eg demographic, clinical, social) and information on exposures and potential confounders | 10, 32-33 |
|  |  |  |  | (b) Indicate number of participants with missing data for each variable of interest | 41 |
|  |  |  |  | (c) Summarise follow-up time (eg, average and total amount) | 31 |
| Outcome data | | 15* | | Report numbers of outcome events or summary measures over time |  |
| Main results | 16 | | (*a*) Give unadjusted estimates and, if applicable, confounder-adjusted estimates and their precision (eg, 95% confidence interval). Make clear which confounders were adjusted for and why they were included | | 34-35 |
|  |  |  | (*b*) Report category boundaries when continuous variables were categorized | | NA |
|  |  |  | (*c*) If relevant, consider translating estimates of relative risk into absolute risk for a meaningful time period | | NA |
| Other analyses | 17 | | Report other analyses done—eg analyses of subgroups and interactions, and sensitivity analyses | | NA |
| **Discussion** | | | | | |
| Key results | 18 | | Summarise key results with reference to study objectives | | 12 |
| Limitations | 19 | | Discuss limitations of the study, taking into account sources of potential bias or imprecision. Discuss both direction and magnitude of any potential bias | | 14-16 |
| Interpretation | 20 | | Give a cautious overall interpretation of results considering objectives, limitations, multiplicity of analyses, results from similar studies, and other relevant evidence | | 12-16 |
| Generalisability | 21 | | Discuss the generalisability (external validity) of the study results | | 15 |
| **Other information** | | | | | |
| Funding | 22 | | Give the source of funding and the role of the funders for the present study and, if applicable, for the original study on which the present article is based | | 21 |
